# Decreasing microbiota-derived uremic toxins to improve CKD outcomes

**DOI:** 10.1093/ckj/sfac154

**Published:** 2022-06-15

**Authors:** Braian M Beker, Iara Colombo, Henry Gonzalez-Torres, Carlos G Musso

**Affiliations:** Human Physiology Department, Instituto Universitario del Hospital Italiano de Buenos Aires, Buenos Aires, Argentina; Human Physiology Department, Instituto Universitario del Hospital Italiano de Buenos Aires, Buenos Aires, Argentina; Facultad de Ciencias de la Salud, Universidad Simón Bolívar, Barranquilla, Colombia; Doctorado en Ciencias Biomédicas, Universidad del Valle, Cali, Valle del Cauca, Colombia; Human Physiology Department, Instituto Universitario del Hospital Italiano de Buenos Aires, Buenos Aires, Argentina; Facultad de Ciencias de la Salud, Universidad Simón Bolívar, Barranquilla, Colombia; Research Department, Hospital Italiano de Buenos Aires, Buenos Aires, Argentina

**Keywords:** butyrate, chronic kidney disease, crotonate, microbiota, probiotic, therapy, uremic toxins

## Abstract

Chronic kidney disease (CKD) is set to become the fifth-leading global cause of death by 2040. This illustrates the many unknowns regarding its pathogenesis and therapy. A key unknown relates to the therapeutic impact of the interaction between CKD and the gut microbiome. The normal gut microbiome is essential for body homeostasis. There is evidence for multiple interactions between the microbiota and CKD—its causes, comorbidities and therapeutic interventions—that are only starting to be unraveled. Thus uremic retention products, such as urea itself, modify the gut microbiota biology and both dietary and drug prescriptions modify the composition and function of the microbiota. Conversely, the microbiota may influence the progression and manifestations of CKD through the production of biologically active compounds (e.g. short-chain fatty acids such as butyrate and crotonate) and precursors of uremic toxins. The present review addresses these issues and their relevance for novel therapeutic approaches ranging from dietary interventions to prebiotics, probiotics, synbiotics and postbiotics, to the prevention of the absorption of microbial metabolites and to increased clearance of uremic toxins of bacterial origin through optimized dialysis techniques or blockade of tubular cell transporters.

## INTRODUCTION

Human microbiota plays a major role in host homeostasis, including micronutrient production, energy metabolism, immune regulation and host defense against pathogens [1, 2]. Additionally, gut microbiota enzymes contribute to the fermentation of ingested nutrients and to bile acid metabolism [2]. In turn, host characteristics, diseases and alimentary habits can have a marked impact on the gut microbiota composition and function. Microbiota changes may affect intestinal transit time and permeability, pH, nutrient assimilation, availability of metabolites that are substrates for the microbiota and produced by the microbiota, including precursors of uremic toxin and potentially chronic kidney disease (CKD) progression or complications [2, 3]. The systemic nature of CKD means that it can also modulate the gut microenvironment and potentially the microbiota [4, 5]. Additionally, spontaneous or prescribed dietary changes and medications can have a profound effect on the microbiome composition in CKD patients [1–3, 5–7]. We now provide an overview of how CKD and its environment (causes, comorbidities, drugs and diet) can influence the microbiota, how the microbiota can modulate CKD and its complications through the production of protective metabolites and uremic toxin precursors and how this knowledge may inform the design of novel therapeutic strategies involving dietary interventions; prebiotics, probiotics and synbiotics; prevention of the absorption of microbial metabolites and increased clearance of uremic toxins of bacterial origin through optimized dialysis techniques or blockade of tubular cell transporters.

## CKD INFLUENCE ON THE GUT MICROBIOTA

There are multiple interactions between CKD and its causes, comorbidities and therapeutic interventions and the microbiota that are only starting to be unraveled.

### Accumulation of metabolites in uremia

The deranged ‘milieu interieur’ in uremia may spill into the gut microbiome environment. High serum urea levels secondary to decreased glomerular filtration increase gastrointestinal tract urea availability. There, microbial urease metabolizes urea into ammonia, which is converted into ammonium hydroxide, increasing the pH of intestinal luminal fluid [1–3]. Ammonia and ammonium hydroxide may promote intestinal epithelial barrier disruption, amplifying systemic inflammation [8]. In addition, ammonia is absorbed and converted back to urea in the liver, explaining the lack of impact on serum urea levels [1, 5]. Consequently, metabolites accumulated in uremia facilitate the growth of microbes that can use these substrates over fiber-consuming bacteria [1]. Constipation (prevalence 29–63% in the CKD population) may increase the growth and replication of these microbes [1, 3]. The net effect is an increased number of bacteria that possess urease, uricase, p-cresol and indole-forming enzymes that boost the production of toxic, pro-inflammatory uremic substances [5]. This potential impact is increased by duodenum and jejunum colonization by aerobic and anaerobic bacteria in uremic patients, as opposed to scarce microbiota in these regions in healthy individuals [5, 9].

Uremic retention solutes may also negatively disrupt the local intestinal mucosal barrier structure. This enables the leakage of bacteria and their metabolites into the endovascular compartment, which is thought to promote systemic inflammation, oxidative stress, and CKD and cardiovascular disease progression [1, 2, 5, 6, 10].

### Causes of CKD and comorbidities

In addition to CKD itself, causes of CKD, such as diabetes, and comorbidities may also be associated with a disrupted microbiota [11].

### Dietary intervention for CKD

In CKD patients, prescribed dietary restriction of potassium can decrease, unintentionally, insoluble fiber consumption, as fruits and vegetables are the main sources of dietary fiber and are frequently limited in low potassium diets [3]. Fiber constitutes the main nutrient for normal colonic bacteria, and decreased fiber availability and fermentation decreases short-chain fatty acids (SCFAs) production [12]. SCFAs are the primary energy sources for colorectal tissues and symbiotic microbes [1, 5, 6, 9, 13]. Additionally, SCFAs (e.g. acetate, butyrate, crotonate) are biologically active by activating specific receptors (e.g. GPR41 and GPR43) and are also involved in posttranslational protein modifications, including histone modifications (e.g. butyrate is an inhibitor of histone deacetylases while crotonate facilitates histone crotonylation), as in epigenetic regulation of gene expression [14–17]. These functions of SCFAs result in anti-inflammatory and nephroprotective functions and may impact embryonic development and future risk of metabolic disease [14–17]. Other dietary interventions prescribed for CKD, such as dietary protein or phosphate restriction, may also modify the microbiota [18].

### Drugs

Antibiotics, which are frequently used in patients with advanced CKD, disrupt the microbiota and this may have long-term consequences for health, especially if recovery of the microbiota is abnormal [19, 20]. Additionally, the interaction of multiple drugs with the microbiota is just beginning to be understood, and includes decreased or increased proliferation of certain bacteria, interference with bacterial enzymatic activities and accumulation and biotransformation of drugs by bacteria, among others [21, 22]. CKD patients are frequently polymedicated and the resulting impact on the healthy gut microbiota as well as the impact of an altered microbiota on prescription drug modification and availability are poorly understood [23].

## INFLUENCE OF MICROBIOTA ON CKD

The gut microbiota processes dietary compounds to generate both metabolites required for human body homeostasis, as exemplified by SCFAs or vitamin K, that may improve CKD and prevent its complications, and precursors of uremic toxins, which may aggravate CKD and its complications [24]. Thus the interaction of diet and microbiota has the potential to both improve and impair the progression and manifestations of CKD. A detailed understanding of these interactions is required to optimize CKD care from the point of view of dietary prescription and of interventions aimed at optimizing the benefit–risk balance of the microbiota–host interaction as it relates to CKD.

Examples of the positive impact of SCFAs were discussed above. Additionally, vitamin K has emerged as a key factor influencing the risk of vascular calcification and death in uremia [25, 26].

Examples of uremic toxins originated from precursors generated by intestinal microbes include indoxyl sulfate (IS), p-cresyl sulfate (pCS), amines, ammonia and trimethylamine N-oxide (TMAO) [10, 27]. IS and pCS are the most studied colon-derived uremic retention solutes, and both have been associated with overall mortality and cardiovascular disease in CKD [6]. More recently, TMAO was shown to contribute to atherosclerosis and CKD progression [2, 28, 29]. As for SCFA production, the amount and types of uremic toxin precursors produced by the microbiota depend on the diet. Thus ingestion of certain metabolites promotes both the growth of bacteria that process them and the generation of uremic toxin precursors. Sources of these metabolites include dietary supplements and red meat or other protein-rich foods, as exemplified by phosphatidylcholine and carnitine for TMA, the precursor of TMAO; tyrosine for p-cresol, the precursor of pCS; and tryptophan for indole, the precursor of IS [24]. Once absorbed, these precursors are metabolized to toxic compounds, mainly by liver cells. Interestingly, the composition of the microbiota may determine the generation of alternative beneficial metabolites from the same precursors. Thus tryptophan may also generate kynurenine pathway metabolites involved in the generation of kidney-protective compounds related to nicotinamide (NAM) adenine dinucleotide (NAD^+^) synthesis [30].

**FIGURE 1:**
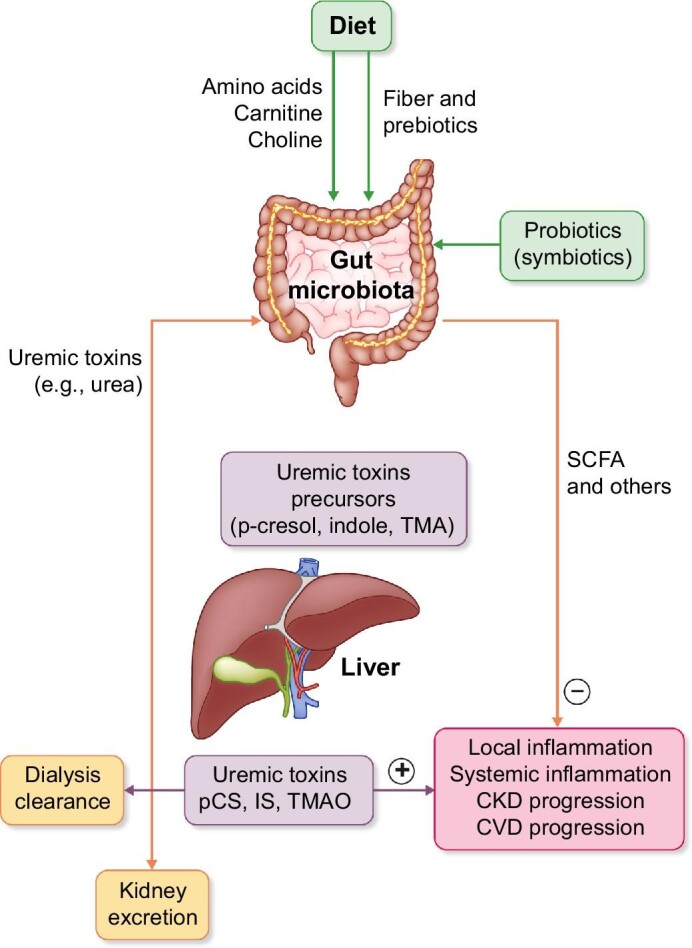
Vicious circle of uremic toxins of intestinal origin and CKD.

The impact of the microbiota composition may be both local, contributing to dampen or promote local inflammation that may further disrupt the intestinal mucosal barrier, and systemic, through the generation of circulating metabolites that protect or damage the kidneys and cardiovascular system (Fig. 1) [5].

## FROM UNDERSTANDING DIET–MICROBIOTA–HOST INTERACTIONS TO OPTIMIZING THERAPY FOR CKD

An improving understanding of the role of an altered gut microbiome in the pathogenesis of systemic inflammation in CKD may lead to the design of novel therapeutic approaches to improve CKD outcomes. These novel approaches aim to restore a healthy gut microbiome to increase the generation of beneficial metabolites and decrease colon-derived uremic solutes. As an alternative, other therapeutic approaches focus on decreasing the absorption or increasing the excretion of microbiota-derived toxins. These interventions include dietary interventions; prebiotics, probiotics and synbiotics; and approaches to decrease metabolite absorption in the gut (e.g. AST-120), to increase dialysis clearance of uremic toxins and to increase the kidney clearance of toxins.

## DIETARY INTERVENTION

Dietary interventions greatly influence the microbiota composition [13, 31]. Diet represents the source of food for both individuals and their microbiota and different bacteria flourish under different food sources. Dietary fiber promotes the growth of bacteria that ferment fiber, which are a source of SCFAs. In contrast, as discussed above, meat-rich diets promote the grow of a different set of bacteria. For example, carnitine-rich diets increase the amount of carnitine-metabolizing bacteria, resulting in lower carnitine bioavailability [32].

Dietary interventions have been tested to reduce the generation of IS and pCS. In another study of 15 vegetarians and 11 omnivores with no prior kidney disease, vegetarians had a 69% higher fiber intake, 25% lower protein intake and markedly lower excretion of pCS and IS (62% and 58%, respectively) [33]. This raises the issue of the relative contribution of low protein versus high fiber to the observation and it is likely that both contribute, low protein by decreasing substrate availability and high fiber by modifying the microbiota and its ability to process the dietary precursors. Fiber consumption, decreasing colonic transit time, may presumably also allow less time for the microbes to break down colonic amino acids to uremic solutes [10, 13, 31]. Thus serum IS but not pCS was significantly reduced within 6 weeks by resistant starch (amylose, a form of digestion resistant fiber) supplementation as compared with digestible starch (amylopectin) in patients on hemodialysis [34]. However, in patients undergoing dialysis, nondigestible carbohydrate supplementation of 10–20 g/day decreased pCS serum levels [10]. In rats, a diet rich in resistant starch (close to 50%) decreased inflammation and oxidative stress and delayed CKD progression compared with rats fed a low fiber diet [35].

Dietary protein restriction has been proposed in CKD to prevent CKD progression and in renal failure to delay the need for dialysis, especially in situations where dialysis access is limited. The aim of the intervention is to reduce the production of nitrogenous waste solutes while also preventing protein malnutrition. It is thought that 0.6–0.8 g/kg/day of protein may achieve this goal, but there is still debate on this [13]. A low protein diet also reduced serum IS levels in predialysis CKD patients [5, 27]. The use of ketoanalogues of amino acids allows a further reduction of dietary protein intake while limiting the negative impact on nitrogen balance. In a controlled crossover study, 32 nondialysis CKD patients (21 males and 11 females) were randomized to either a very low protein diet (0.3 g/kg/day) supplemented with ketoanalogues during the first week and a low protein diet during the second week (*n* = 16) or a low protein diet during the first week and a very low protein diet supplemented with ketoanalogues during the second week (*n* = 16). The very low protein diet supplemented with ketoanalogues was associated with a significant reduction of serum IS [36].

Despite these promising results, it remains unclear to what extent dietary interventions aimed at modulating the microbiota and its production of SCFAs or uremic toxin precursor results in improved long-term outcomes. The recent availability of safer and better-tolerated potassium binders such as patiromer and sodium zirconium cyclosilicate will allow us to test the hypothesis that increasing potassium (and fiber) intake will contribute to a higher fiber content of the diet and improved microbiota profile [37, 38]. While clinical trials are under way to test the feasibility of the approach and also to test the impact on CKD progression or other outcomes (e.g. NCT05056727, NCT04207203), the design should be very precise to differentiate any impact on the microbiota from the impact of more prolonged use or a higher dose of renin–angiotensin system blockers or mineralocorticoid receptor antagonists.

## PREBIOTICS, PROBIOTICS, SYNBIOTICS AND POSTBIOTICS

Prebiotics and probiotics may influence the composition of intestinal microbiota. Prebiotics are compounds in food that induce the growth or activity of beneficial microorganisms while probiotics refer to living microorganisms that, if ingested in adequate amounts, will increase favorable bacteria by improving the intestinal microbiota profile. Synbiotics are a mixture of probiotics and prebiotics that beneficially affect the host by promoting the survival and activity of beneficial microorganisms. Postbiotics are inanimate microorganisms and/or their components that confer a health benefit on the host. Postbiotics must contain inactivated microbial cells or cell components, with or without metabolites, that contribute to observed health benefits [39]. However, isolated metabolites are not considered postbiotics.

Therapeutic approaches based on prebiotics, probiotics or synbiotics may be helpful for CKD, but there is much less information on postbiotics, given the recent development of a consensus definition and the fact that older literature on postbiotic may not actually refer to the current concept of postbiotics [39]. A recent meta-analysis evaluated the effects of probiotic, prebiotic and synbiotic supplements on systemic inflammatory biomarkers [i.e. C-reactive protein (CRP)], oxidative stress status [i.e. malondialdehyde (MDA)], total antioxidant capacity (TAC), total glutathione (GSH)] and on the lipid profile [i.e. triglycerides (TG), total cholesterol (TC), low-density lipoprotein (LDL) cholesterol and high-density lipoprotein (HDL) cholesterol] among patients with CKD. The study found that prebiotics, probiotics and synbiotics reduced CRP levels, increased antioxidant enzymes (TAC, GSH) while decreasing oxidative (MDA) activity and ameliorated the lipid profile (TC, HDL and LDL) among individuals with CKD compared with control groups. The use of synbiotics had a larger effect on these indicators compared with the isolated use of probiotics or prebiotics [40].

### Probiotics

*Lactobacillus*, *Streptococcus* and *Bifidobacterium* are the most widely used probiotics [3]. Recently, *Lactobacillus casei* Zhang was shown to prevent kidney injury in experimental animals, a pilot human study showed promising results and was shown to have antiobesity effects [41, 42]. Lebenin, an antibiotic-resistant lactic acid bacteria oral preparation or *Bifidobacterium* oral capsules reduced serum IS levels in hemodialysis patients [43, 44]. *Lactobacillus* in hemodialysis patients has demonstrated a reduction in small intestine toxins levels (dimethylamine and nitrosodimethylamine) while bifidobacteria, along with a reduction in serum IS levels, have also exhibited a decrease in homocysteine and TG. Studies have shown improvements in symptoms and quality of life in patients with advanced CKD after 6 months of probiotic supplementation [2]. It has been proposed that toxic microbial metabolite production can be modulated by increasing saccharolytic and reducing proteolytic bacteria in the colon [6]. However, the mere addition of probiotics to the diet, in the absence of other dietary changes that promote the survival of ingested probiotics, is unlikely to restore normal composition, structure and function of gut microbiota in CKD, and negative results have also been reported [1, 5].

### Prebiotics

Only bifidogenic, nondigestible oligosaccharides such as inulin, oligofructose and galacto-oligosaccharides fulfil all the prebiotic criteria [2, 3, 6]. Fermentable carbohydrates shifted nitrogen excretion from the urinary tract to fecal excretion, reducing serum urea concentrations in patients with CKD [3]. The prebiotic arabino-xylo-oligosaccharide improved insulin resistance and dyslipidemia in mice with CKD [45]. In chronic hemodialysis patients, an increasing dose of oligofructose-enriched inulin reduced serum pCS levels and generation rates at 1 month but serum IS levels and IS generation rates remained unchanged [46].

### Synbiotics

Synbiotics have been reported to normalize bowel habits in CKD patients while reducing pCS and IS serum concentrations [1, 2, 47]. In a randomized, double-blind, placebo-controlled, crossover trial in 37 predialysis CKD patients over 6 weeks, symbiotic therapy (prebiotic component: inulin, fructo-oligosaccharides, and galacto-oligosaccharides; probiotic component: nine different strains across the *Lactobacillus*, *Bifidobacterium*, and *Streptococcus* genera) decreased serum pCS and favorably modified the stool microbiome but did not modify serum IS. Interestingly, serum pCS and IS decreased more in patients who did not receive antibiotics during the study [48].

## PREVENTING THE ABSORPTION OF MICROBIAL METABOLITES

AST-120 is an oral sorbent composed of spherical porous carbon particles. It adsorbs small organic molecules that may accumulate in CKD patients [27]. In CKD patients and rats, AST-120 reduced serum and urine IS through adsorption of its precursor indole in the gut, resulting in a shifting from absorption in the gut to excretion in feces [27, 31, 47]. It is currently in clinical use in Japan to decrease the absorption of uremic toxins such as indols and retard CKD. However, two large multinational trials (EPPIC-1 and EPPIC-2) failed to show a benefit on CKD progression [49]. Potential reasons for the failure to demonstrate efficacy outside Japan include the large pill burden.

## OPTIMIZED EXTRACORPOREAL CLEARANCE

Hemodialysis fails to optimally clear many uremic toxins originated in the gut microbiota, as they are protein bound. Examples include IS and pCS, among others [2, 6, 27, 31, 36]. Strategies are being developed focused on increasing removal of these metabolites, however, none are used routinely in the clinic. These strategies include combining high-volume ultrafiltration with hemodialysis and the use of sorbents [10, 31, 50], in addition to the arterial bloodline molecules that compete with uremic toxins for protein binding [27, 51].

## INTERFERENCE WITH TUBULAR CELL TRANSPORTERS

Active renal tubular secretion through cell membrane transporters is the main route for excretion of protein-bound toxins excretion. Multiple transporters contribute to this task, but organic anion transporters 1 and 3 (OAT1 and OAT3) have been studied in most detail. OAT1 and OAT3 are located on the basolateral membrane of tubular cells and transport toxins such as IS into tubular cells. OAT1 is present in proximal tubular cells and OAT3 is also present in distal tubular cells. OAT4 in the apical membrane of tubular cells secretes IS into the urinary space [52]. *In vitro* studies performed in rats showed that inhibiting the influx of IS into proximal tubular cells by blocking OAT1 by probenecid increased cell viability. According to the authors, this suggests that accumulation of IS within tubular cells may accelerate tubulointerstitial damage, resulting in nephrotoxicity [53]. Another *in vivo* experiment performed in mice concluded that both transporters (OAT1 and OAT3) contribute to the uptake and elimination of uremic toxins. While inhibiting them may potentially diminish CKD progression, it may lead to a significant level of uremic toxins accumulation in the plasma, increasing systemic exposure to them [53]. No study to date has tested the effect of OAT1 and OAT3 transporters in uremic toxins clearance in humans. Further research is needed to understand the potential benefits of inhibiting the transporters to delay CKD progression or the consequences of doing so.

## CONCLUSION

There is a growing certainty that the interaction of diet, microbiota and CKD contributes to CKD progression and to CKD complications. This has led to the development of therapeutic approaches aimed at optimizing the diet–microbiota interaction to modulate the gut microbiota towards a healthy phenotype that decreases CKD progression and its cardiovascular impact. As alternatives, preventing the absorption of microbiota-generated toxin precursors or increasing the excretion of these toxins is being explored. So far, steps have been taken in the right direction but have not yet permeated to general clinical practice. Before novel therapeutic interventions reach clinical practice, clinical trials should demonstrate that the approach is feasible, safe and effective in improving key outcomes in the long term. This will need a considerable investment in terms of funding and research efforts.

## CONFLICT OF INTEREST STATEMENT

All the authors declare that they have no conflicts of interest.

## References

[1] Vaziri ND. Effect of synbiotic therapy on gut–derived uremic toxins and the intestinal microbiome in patients with CKD. Clin J Am Soc Nephrol 2016; 11: 199–201 26772192 10.2215/CJN.13631215 PMC4741052

[2] Mafra D , Lobo JC, Barros AF et al. Role of altered intestinal microbiota in systemic inflammation and cardiovascular disease in chronic kidney disease. Future Microbiol 2014;9: 399–410 24762311 10.2217/fmb.13.165

[3] Evenepoel P , Meijers BKI, Bammens BRM et al. Uremic toxins originating from colonic microbial metabolism. Kidney Int 2009; 76(Suppl 114): S12–S19 10.1038/ki.2009.402 19946322

[4] Zoccali C , Vanholder R, Massy ZA et al. The systemic nature of CKD. Nat Rev Nephrol 2017; 13: 344–358 28435157 10.1038/nrneph.2017.52

[5] Pahl MV , Vaziri ND. The chronic kidney disease-colonic axis. Semin Dial 2015; 28: 459–463 25855516 10.1111/sdi.12381

[6] Poesen R , Meijers B, Evenepoel P. The colon: an overlooked site for therapeutics in dialysis patients. Semin Dial 2013; 26: 323–332 23458264 10.1111/sdi.12082

[7] Castillo-Rodriguez E , Fernandez-Prado R, Esteras R et al. Impact of altered intestinal microbiota on chronic kidney disease progression. Toxins 2018; 10: E300 30029499 10.3390/toxins10070300 PMC6070989

[8] Vaziri ND , Yuan J, Norris K. Role of urea in intestinal barrier dysfunction and disruption of epithelial tight junction in chronic kidney disease. Am J Nephrol 2013; 37: 1–6 23258127 10.1159/000345969 PMC3686571

[9] Ritz E. Intestinal-renal syndrome: mirage or reality? Blood Purif 2011; 31: 70–76 21228570 10.1159/000321848

[10] Meyer TW , Hostetter TH. Uremic solutes from colon microbes. Kidney Int 2012; 81: 949–954 22318422 10.1038/ki.2011.504

[11] Tilg H , Moschen AR. Microbiota and diabetes: an evolving relationship. Gut 2014; 63: 1513–1521 24833634 10.1136/gutjnl-2014-306928

[12] Makki K , Deehan EC, Walter J et al. The impact of dietary fiber on gut microbiota in host health and disease. Cell Host Microbe 2018; 23: 705–715 29902436 10.1016/j.chom.2018.05.012

[13] Sirich TL. Dietary protein and fiber in end stage renal disease. Semin Dial 2015; 28: 75–80 25319504 10.1111/sdi.12315

[14] Fontecha-Barriuso M , Martin-Sanchez D, Ruiz-Andres O et al. Targeting epigenetic DNA and histone modifications to treat kidney disease. Nephrol Dial Transplant 2018; 33: 1875–1886 29534238 10.1093/ndt/gfy009

[15] Ruiz-Andres O , Sanchez-Niño MD, Cannata-Ortiz P et al. Histone lysine crotonylation during acute kidney injury in mice. Dis Model Mech 2016; 9: 633–645 27125278 10.1242/dmm.024455 PMC4920150

[16] Sanchez-Niño MD , Aguilera-Correa J-J, Politei J et al. Unraveling the drivers and consequences of gut microbiota disruption in Fabry disease: the lyso-Gb3 link. Future Microbiol 2020; 15: 227–231 32271110 10.2217/fmb-2019-0249

[17] Kimura I , Miyamoto J, Ohue-Kitano R et al. Maternal gut microbiota in pregnancy influences offspring metabolic phenotype in mice. Science 2020; 367: eaaw8429 32108090 10.1126/science.aaw8429

[18] Favero C , Carriazo S, Cuarental L et al. Phosphate, microbiota and CKD. Nutrients 2021; 13: 1273 33924419 10.3390/nu13041273 PMC8070653

[19] Lynn MA , Eden G, Ryan FJ et al. The composition of the gut microbiota following early-life antibiotic exposure affects host health and longevity in later life. Cell Rep 2021; 36: 109564 34433065 10.1016/j.celrep.2021.109564

[20] Maier L , Goemans CV, Wirbel J et al. Unravelling the collateral damage of antibiotics on gut bacteria. Nature 2021; 599: 120–124 34646011 10.1038/s41586-021-03986-2 PMC7612847

[21] Klünemann M , Andrejev S, Blasche S et al. Bioaccumulation of therapeutic drugs by human gut bacteria. Nature 2021; 597: 533–538 34497420 10.1038/s41586-021-03891-8 PMC7614428

[22] Maier L , Pruteanu M, Kuhn M et al. Extensive impact of non-antibiotic drugs on human gut bacteria. Nature 2018; 555: 623–628 29555994 10.1038/nature25979 PMC6108420

[23] van Oosten MJM , Logtenberg SJJ, Hemmelder MH et al. Polypharmacy and medication use in patients with chronic kidney disease with and without kidney replacement therapy compared to matched controls. Clin Kidney J 2021; 14: 2497–2523 34950462 10.1093/ckj/sfab120 PMC8690067

[24] Fernandez-Prado R , Esteras R, Perez-Gomez MV et al. Nutrients turned into toxins: microbiota modulation of nutrient properties in chronic kidney disease. Nutrients 2017; 9: E489 28498348 10.3390/nu9050489 PMC5452219

[25] Levy DS , Grewal R, Le TH. Vitamin K deficiency: an emerging player in the pathogenesis of vascular calcification and an iatrogenic consequence of therapies in advanced renal disease. Am J Physiol Renal Physiol 2020; 319: F618–F623 32830534 10.1152/ajprenal.00278.2020

[26] Shea MK , Barger K, Booth SL et al. Vitamin K status, all-cause mortality, and cardiovascular disease in adults with chronic kidney disease: the chronic renal insufficiency cohort. Am J Clin Nutr 2022; 115: 941–948 10.1093/ajcn/nqab375 PMC8895220 34788785

[27] Niwa T. Targeting protein-bound uremic toxins in chronic kidney disease. Expert Opin Ther Targets 2013; 17: 1287–1301 23941498 10.1517/14728222.2013.829456

[28] Tang WHW , Wang Z, Levison BS et al. Intestinal microbial metabolism of phosphatidylcholine and cardiovascular risk. N Engl J Med 2013; 368: 1575–1584 23614584 10.1056/NEJMoa1109400 PMC3701945

[29] Tang WHW , Wang Z, Kennedy DJ et al. Gut microbiota-dependent trimethylamine N-oxide (TMAO) pathway contributes to both development of renal insufficiency and mortality risk in chronic kidney disease. Circ Res 2015; 116: 448–455 25599331 10.1161/CIRCRESAHA.116.305360 PMC4312512

[30] Fontecha-Barriuso M , Lopez-Diaz AM, Carriazo S et al. Nicotinamide and acute kidney injury. Clin Kidney J 2021; 14: 2453–2462 34950458 10.1093/ckj/sfab173 PMC8690056

[31] Leong S , Sirich T. Indoxyl sulfate—review of toxicity and therapeutic strategies. Toxins 2016; 8: 358 10.3390/toxins8120358 PMC5198552 27916890

[32] Sanchez-Niño MD , Ortiz A. Differential effects of oral and intravenous L-carnitine on serum lipids: is the microbiota the answer? Clin Kidney J 2014; 7: 437–441 25878774 10.1093/ckj/sfu099 PMC4379349

[33] Patel KP , Luo FJ-G, Plummer NS et al. The production of p-cresol sulfate and indoxyl sulfate in vegetarians versus omnivores. Clin J Am Soc Nephrol 2012; 7: 982–988 22490877 10.2215/CJN.12491211 PMC3362314

[34] Sirich TL , Plummer NS, Gardner CD et al. Effect of increasing dietary fiber on plasma levels of colon-derived solutes in hemodialysis patients. Clin J Am Soc Nephrol 2014; 9: 1603–1610 25147155 10.2215/CJN.00490114 PMC4152802

[35] Vaziri ND , Liu S-M, Lau WL et al. High amylose resistant starch diet ameliorates oxidative stress, inflammation, and progression of chronic kidney disease. PLoS One 2014; 9: e114881 25490712 10.1371/journal.pone.0114881 PMC4260945

[36] Marzocco S , Dal Piaz F, Di Micco L et al. Very low protein diet reduces indoxyl sulfate levels in chronic kidney disease. Blood Purif 2013; 35: 196–201 23485887 10.1159/000346628

[37] Valdivielso JM , Balafa O, Ekart R et al. Correction to: Hyperkalemia in chronic kidney disease in the new era of kidney protection therapies. Drugs 2021; 81: 1819 34633647 10.1007/s40265-021-01617-8

[38] Valdivielso JM , Balafa O, Ekart R et al. Hyperkalemia in chronic kidney disease in the new era of kidney protection therapies. Drugs 2021; 81: 1467–1489 34313978 10.1007/s40265-021-01555-5

[39] Salminen S , Collado MC, Endo A et al. The International Scientific Association of Probiotics and Prebiotics (ISAPP) consensus statement on the definition and scope of postbiotics. Nat Rev Gastroenterol Hepatol 2021; 18: 649–667 33948025 10.1038/s41575-021-00440-6 PMC8387231

[40] Zheng HJ , Guo J, Wang Q et al. Probiotics, prebiotics, and synbiotics for the improvement of metabolic profiles in patients with chronic kidney disease: a systematic review and meta-analysis of randomized controlled trials. Crit Rev Food Sci Nutr 2021; 61: 577–598 32329633 10.1080/10408398.2020.1740645

[41] Zhu H , Cao C, Wu Z et al. The probiotic *L. casei* Zhang slows the progression of acute and chronic kidney disease. Cell Metab 2021; 33: 1926–1942.e8 34270930 10.1016/j.cmet.2021.06.014

[42] He Q , Zhang Y, Ma D et al. *Lactobacillus casei* Zhang exerts anti-obesity effect to obese glut1 and gut-specific-glut1 knockout mice via gut microbiota modulation mediated different metagenomic pathways. Eur J Nutr 2022; 61: 2003–2014 34984487 10.1007/s00394-021-02764-0

[43] Hida M , Aiba Y, Sawamura S et al. Inhibition of the accumulation of uremic toxins in the blood and their precursors in the feces after oral administration of Lebenin, a lactic acid bacteria preparation, to uremic patients undergoing hemodialysis. Nephron 1996; 74: 349–355 8893154 10.1159/000189334

[44] Takayama F , Taki K, Niwa T. *Bifidobacterium* in gastro-resistant seamless capsule reduces serum levels of indoxyl sulfate in patients on hemodialysis. Am J Kidney Dis 2003; 41(3Suppl 1): S142–S145 12612972 10.1053/ajkd.2003.50104

[45] Koppe L , Pillon NJ, Vella RE et al. p-Cresyl sulfate promotes insulin resistance associated with CKD. J Am Soc Nephrol 2013; 24: 88–99 23274953 10.1681/ASN.2012050503 PMC3537215

[46] Meijers BKI , De Preter V, Verbeke K et al. p-Cresyl sulfate serum concentrations in haemodialysis patients are reduced by the prebiotic oligofructose-enriched inulin. Nephrol Dial Transplant 2010; 25: 219–224 19692415 10.1093/ndt/gfp414

[47] Neirynck N , Glorieux G, Schepers E et al. Review of protein-bound toxins, possibility for blood purification therapy. Blood Purif 2013; 35(Suppl 1): 45–50 23466378 10.1159/000346223

[48] Rossi M , Johnson DW, Morrison M et al. Synbiotics Easing Renal Failure by Improving Gut Microbiology (SYNERGY): a randomized trial. Clin J Am Soc Nephrol 2016; 11: 223–231 26772193 10.2215/CJN.05240515 PMC4741035

[49] Schulman G , Berl T, Beck GJ et al. Randomized placebo-controlled EPPIC trials of AST-120 in CKD. J Am Soc Nephrol 2015; 26: 1732–1746 25349205 10.1681/ASN.2014010042 PMC4483576

[49] Paats J , Adoberg A, Arund J et al. Serum levels and removal by haemodialysis and haemodiafiltration of tryptophan-derived uremic toxins in ESKD patients. Int J Mol Sci 2020; 21: E1522 32102247 10.3390/ijms21041522 PMC7073230

[50] Madero M , Cano KB, Campos I et al. Removal of protein-bound uremic toxins during hemodialysis using a binding competitor. Clin J Am Soc Nephrol 2019; 14: 394–402 30755453 10.2215/CJN.05240418 PMC6419294

[51] Taki K , Nakamura S, Miglinas M et al. Accumulation of indoxyl sulfate in OAT1/3-positive tubular cells in kidneys of patients with chronic renal failure. J Ren Nutr 2006; 16: 199–203 16825019 10.1053/j.jrn.2006.04.020

[52] Enomoto A , Takeda M, Tojo A et al. Role of organic anion transporters in the tubular transport of indoxyl sulfate and the induction of its nephrotoxicity. J Am Soc Nephrol 2002; 13: 1711–1720 12089366 10.1097/01.asn.0000022017.96399.b2

[53] Wu W , Bush KT, Nigam SK. Key role for the organic anion transporters, OAT1 and OAT3, in the in vivo handling of uremic toxins and solutes. Sci Rep 2017; 7: 4939 28694431 10.1038/s41598-017-04949-2 PMC5504054

